# Appetite Enhancement and Weight Gain by Peripheral Administration of TrkB Agonists in Non-Human Primates

**DOI:** 10.1371/journal.pone.0001900

**Published:** 2008-04-02

**Authors:** John C. Lin, David Tsao, Paul Barras, Raul A. Bastarrachea, Bob Boyd, Joyce Chou, Rodnie Rosete, Hua Long, Alison Forgie, Yasmina Abdiche, Jeanette Dilley, Jennifer Stratton, Carlos Garcia, David L. Sloane, Anthony G. Comuzzie, Arnon Rosenthal

**Affiliations:** 1 Rinat, Pfizer Inc., South San Francisco, California, United States of America; 2 Southwest Foundation for Biomedical Research, San Antonio, Texas, United States of America; 3 Alpha Genesis, Inc., Yemassee, South Carolina, United States of America; 4 Northern Biomedical Research, Muskegon, Michigan, United States of America; Cambridge University, United Kingdom

## Abstract

Loss of function mutations in the receptor tyrosine kinase TrkB pathway resulted in hyperphagia and morbid obesity in human and rodents. Conversely, peripheral or central stimulation of TrkB by its natural ligands BDNF or NT4 reduced body weight and food intake in mice, supporting the idea that TrkB is a key anorexigenic signal downstream of the melanocortin-4 receptor (Mc4r) system. Here we show that in non-human primates TrkB agonists were anorexigenic when applied centrally, but surprisingly orexigenic, leading to gain in appetite, body weight, fat deposits and serum leptin levels, when given peripherally. The orexigenic and pro-obesity effects of peripherally administered TrkB agonists appear to be dose dependent, not associated with fluid retention nor with evidence of receptor down regulation. Our findings revealed that TrkB signaling exerts dual control on energy homeostasis in the primates that could be targeted for the treatment of either wasting disorders or obesity.

## Introduction

Brain-derived neurotrophic factor (BDNF) and neurotrophin-4 (NT4) are two naturally occurring ligands for the receptor tyrosine kinase trkB [1]. Originally viewed as trophic factors for neuronal survival and neurite outgrowth during embryonic development, these factors can actually exert a wide range of biological functions in the adult, such as long term potentiation and synaptic plasticity. The mRNA of BDNF is normally expressed in the ventromedial hypothalamus (VMH) [2], [3], [4], [5]. The VMH expression of BDNF mRNA is reduced under several conditions where the appetite is increased, such as food deprivation, melanocortin antagonism (as in *A^y^* lethal yellow mice) and genetic ablation of melanocortin 4 receptor (Mc4r) [3].

The loss-of-function mutations of BDNF or trkB loci in mice led to a syndrome of hyperphagia and obesity. These include mice heterozygous with a BDNF deficient allele [2], [4], mice with postnatal brain-specific BDNF deletion [5], as well as mice with a hypomorphic allele of trkB [3]. Remarkably a *de novo* trkB mutation was identified in a mentally retarded, morbidly obese child [6]. The kinase activity of this mutant human trkB allele is greatly diminished. In addition, a human case of hyperphagia and obesity was found to harbor a chromosomal translocation affecting BDNF expression [7]. Furthermore, both central and peripheral administration of various TrkB agonists suppressed food intake and body weight in several mouse models of obesity [2], [3], [8], [9]. These findings together support the notion that trkB activation by BDNF expressed in the brain is essential for appetite regulation and energy homeostasis.

To investigate the feasibility of trkB agonism as a therapeutic approach for human obesity, we conducted a series of experiments using NT4, BDNF and TrkB agonistic antibody in several species of non-human primates. Both NT4 and BDNF, when delivered into the brain directly, suppressed food consumption in the lean monkeys. Contrary to our expectation, however, NT4 and TrkB agonistic antibody significantly increased food intake, body weight, fat mass and circulating leptin levels in the lean monkeys and even in the obese baboons. Further analysis suggested a novel, peripherally accessible, orexigenic TrkB system, which when activated can counter-balance the central, anorexigenic TrkB system.

## Results

### Administration of TrkB Agonists Result in Hypophagia and Weight Loss in Mice

**Figure 1:**
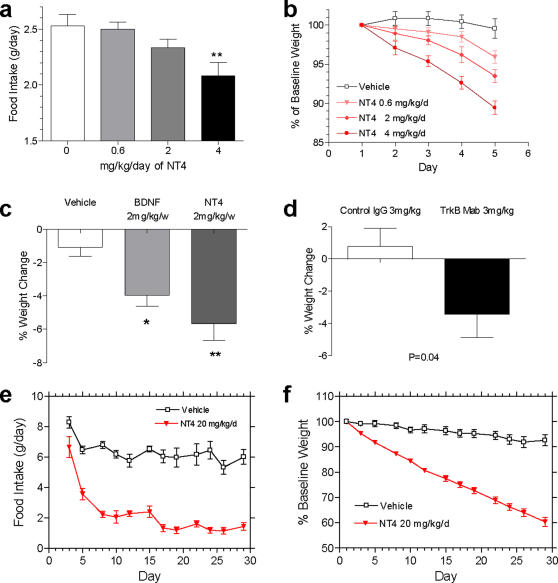
Treatment with TrkB agonists reduces food intake and weight in mice. a, Daily IV injection of 0.6, 2 or 4 mg/kg of NT4 (n = 6 per group) resulted in dose dependent reduction in average daily food intake over 5 days in adult male DIO mice (One way ANOVA, P = 0.0098, F = 4.967 with Dunnett's posttest, P<0.01 for 4 mg/kg group compared to the vehicle group, while no statistically significant effects were observed at 0.6 or 2 mg/kg). b, Daily IV injection of 0.6, 2 or 4 mg/kg of NT4 (n = 6 per group) resulted in dose dependent reduction in body weight in adult male DIO mice (Two way ANOVA, P<0.0001 with Bonferroni's posttests, data are presented as % change relative to baseline body weight). c, Weekly IV injection of 2 mg/kg BDNF (n = 7) or NT4 (n = 7) led to significant weight loss compared to the vehicle treatment (n = 8) in adult male DIO mice at 10 days after the first dose (One way ANOVA, P = 0.002, F = 9.08 with Dunnett's posttest). d, Single IV injection of 3 mg/kg TrkB agonist monoclonal antibody (n = 7) significantly reduced body weight relative to control IgG treatment (n = 7) in adult male DIO mice 10 days after dosing (Student's t test, 2 tailed, P = 0.04). e–f, Daily treatment of Leptin receptor-deficient, 12 week old female *db/db* mice for 30 days with IV-delivered 20 mg/kg of NT4 (n = 7) decreased daily food intake by 50–80% (e), and body weight by 40% (f) relative to the vehicle treated mice (n = 8).

Since NT4 has very similar agonist profile as BDNF [10], [11], [12], we use these two factors separately and interchangeably as the naturally occurring, TrkB agonist agents. Daily intravenous (IV) treatment of diet induced obesity (DIO) mouse model with 0.6, 2 or 4 mg/kg of NT4 led to a dose dependent reduction in food intake (Fig. 1a) and body weight (Fig. 1b). Weight reduction was also observed at Day 11 following weekly IV injections of 2mg/kg of BDNF, 2mg/kg of NT4 (Fig. 1c) or a single IV injection of 3mg/kg of a TrkB selective agonist antibody (Fig. 1d; Tsao et al, 2007; for more details on the generation and characterization of the TrkB agonist antibody see also Supplemental Methods S1, Supplemental Figs S1–S2 and Supplemental Table S1). Daily subcutaneous (SC) treatment of 12 weeks old obese female *db/db* mice with 20 mg/kg of NT4 (n = 8 per group) for 30 days also led to a sustained 50–80% decrease in daily food intake (Fig. 1e), and a linearly time-dependent, 40% decrease in body weight (Fig. 1f), confirming that activation of TrkB alone causes anorexia and weight loss in rodents independent of the leptin signal [9]. No tolerance, desensitization, adaptation or resistance to the extended exposure to high dose of NT4 was evident under these treatment conditions.

### Central Administration of TrkB Agonists Suppress Food Intake in Monkeys

**Figure 2:**
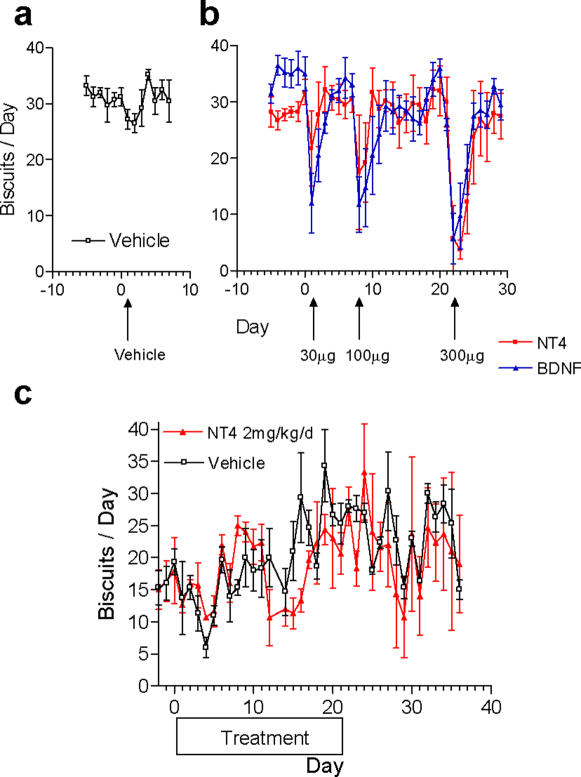
Centrally, but not peripherally, delivered TrkB agonists suppress food intake in Rhesus monkeys. a, Vehicle infusion into normal adult Rhesus monkeys (baseline body weight 4–7 kg) via an ICV catheter did not substantially change the food consumption (n = 2 males and 2 females per group). b, Dose dependent and reversible decrease in food intake followed ICV infusion into normal adult Rhesus monkeys of increasing doses of NT4 or BDNF (n = 2 males and 2 females per group). c , Daily SC injections of 2 mg/kg NT4 (n = 1 male and 2 females per group) over 21 days in lean, adult Rhesus monkeys (baseline body weight 3–6 kg) did not significantly alter the level of daily food intake.

Similar to the findings with central administration of TrkB agonists in mice [2], [8], [9], an anorexigenic effect was observed in adult Rhesus monkeys that received intracerebroventricular (ICV) injection of BDNF or NT4 (30–300 µg) compared to the vehicle injection (Figs 2a, 2b). The reduction in food intake after ICV injections in the Rhesus was fully reversible within 3–4 days and was not accompanied by signs of inflammation, such as fever or leukocytosis (data not shown). Together these data established that TrkB activation in the central nervous system mediates a robust anorexigenic signal in both mice and non-human primates.

### Peripheral Administration of TrkB Agonists Lead to Hyperphagia and Weight Gain in Monkeys

**Figure 3:**
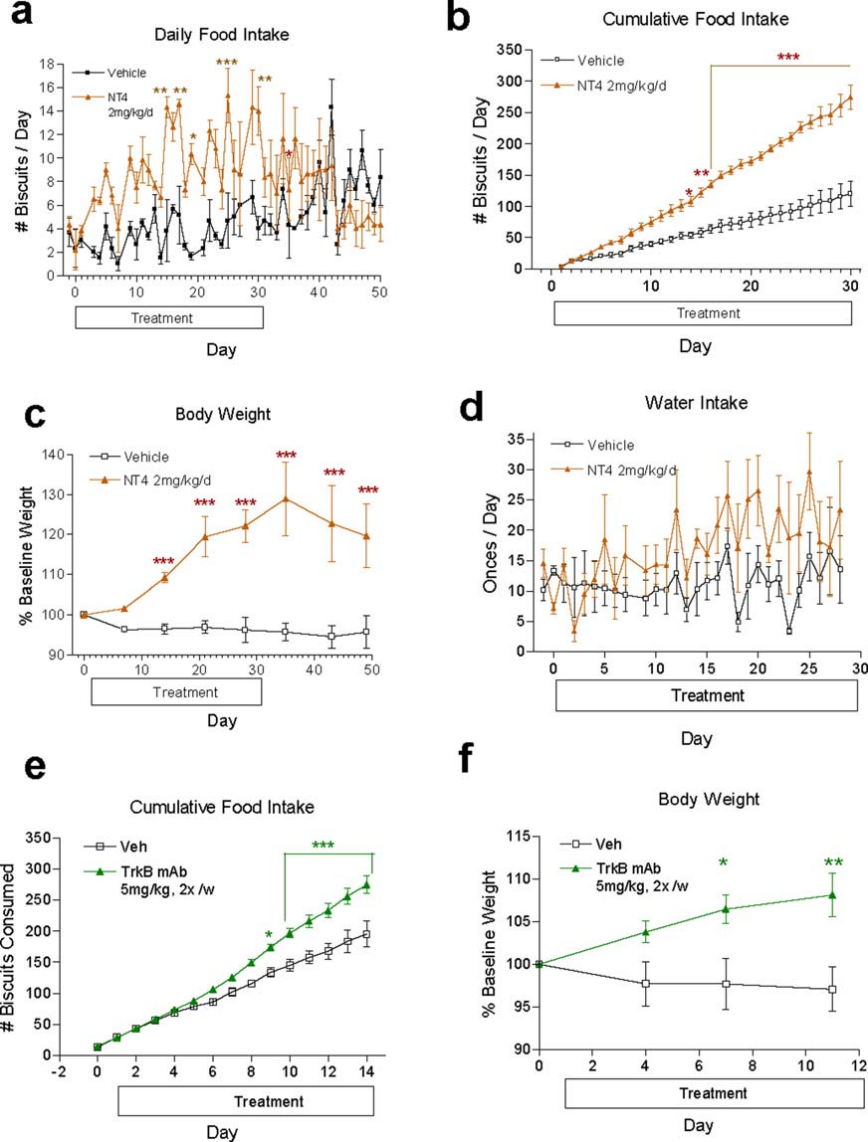
Peripheral application of TrkB agonists leads to gain in appetite, fat deposits and weight in lean Cynomolgus monkeys. a–d, Daily treatment of non-obese, adult females, Cynomolgus (baseline body weight 3–5 kg) with 2 mg/kg/day NT4 IV (n = 3 per group) for 30 days led to a significant increase in daily food intake (a), cumulative food intake (b) and body weight (c) without significant change in water intake (d). e–f, Lean, adult female Cynomolgus monkeys (n = 3 per group) that were treated twice a week with IV injections of 5 mg/kg of a TrkB agonist antibody exhibited a significant increase in cumulative food intake (e) and weight gain (f)

Unlike the central delivery of TrkB agonists, daily SC administration of NT4 at 2 mg/kg in lean adult Rhesus monkeys over 21 days led to 10–15% body weight gain relative to the vehicle group (n = 1 male and 2 females per group) without significantly affecting daily food intake (Fig. 2c and data not shown). Moreover, lean adult female Cynomolgus monkeys that received daily SC (4 hour post-dosing serum NT4 levels at 600–1200 ng/mL) or IV (peak serum NT4 levels at 5–12 µg/mL) injections of 2 mg/kg NT4 for 21 or 30 days displayed 2 to 3-fold increase in daily food intake (Fig. 3a and Supplemental Fig. S3a) and 1.6 to 2.3-fold increase in cumulative food intake respectively (Fig. 3b and Supplemental Fig. S3b). Food intake reverted to normal levels within 10 days of dosing termination. Consequently, such daily SC or IV administrations of NT4 resulted in a 16% (SC for 21 days) or 33% (IV for 30 days) increase of body weight, respectively (Fig. 3c and Supplemental Fig. S3c). These results revealed a robust pro-obesity and orexigenic effect following peripheral delivery of NT4 in the non-human primates. No significant changes in water intake (Fig. 3d), menstrual cycle, peripheral blood cell counts, insulin, C peptide, triglycerides, total cholesterol, HDL, LDL, free fatty acids, or electrolytes were detected in the Cynomolgus monkeys (Supplemental Figs. S4, S5, S6, and data not shown). These results indicate that, in general, peripheral administration of NT4 was well tolerated and that the weight gain was not accompanied by hemodilution or fluid retention.

To test whether TrkB activation alone is sufficient to induce obesity and appetite, lean, adult female Cynomolgus monkeys were treated twice a week with IV injections of 5 mg/kg of the TrkB agonist antibody. These monkeys also exhibited 40% increase in cumulative food intake (Fig. 3e) and 10% increase in body weight (Fig. 3f) within 2 weeks. Thus activation of the TrkB tyrosine kinase receptor by peripheral administration of a natural agonist, NT4, or by a TrkB agonist antibody, leads to potent orexigenic and pro-obesity effects in primates.

### Peripheral NT4 Injections Increase Food Consumption, Body Weight, Fat Mass and Circulating Leptin in Obese Baboons

**Figure 4:**
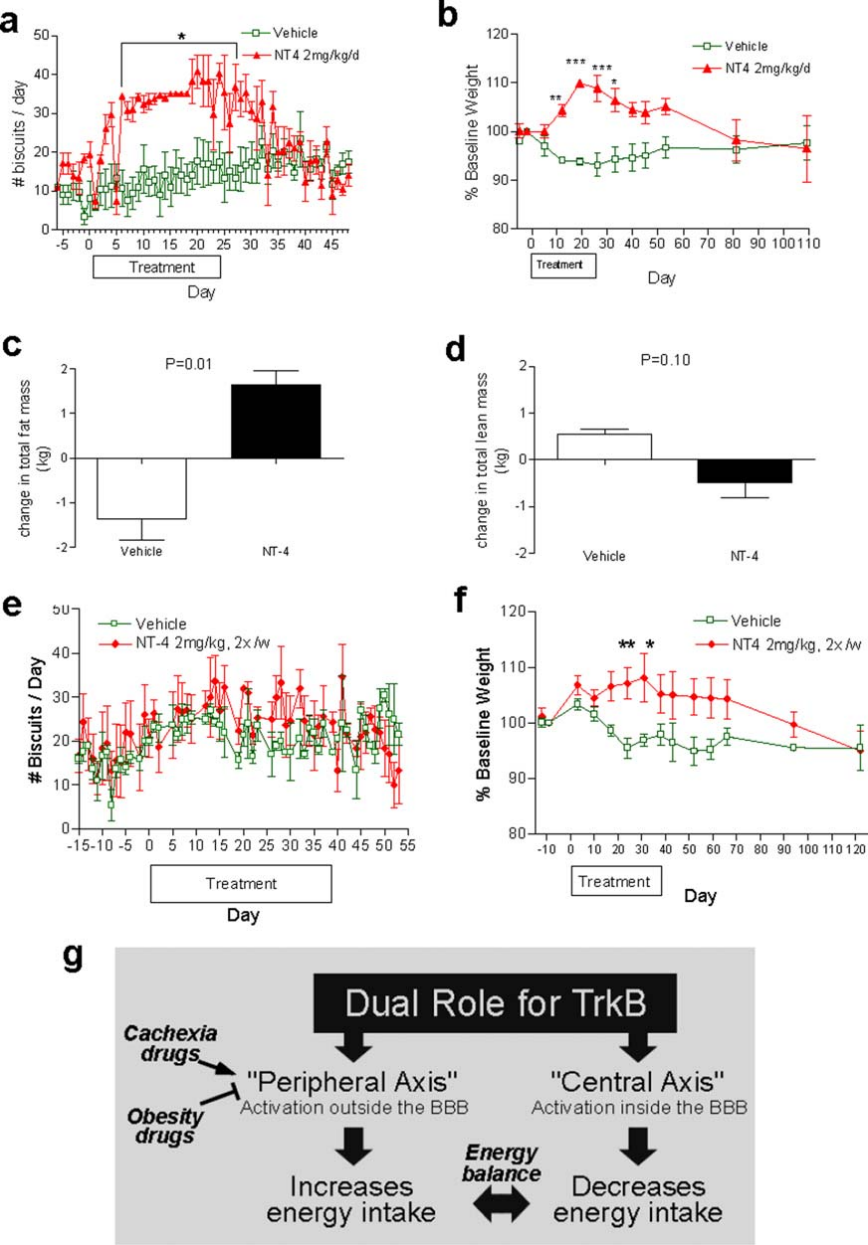
Effects of TrkB agonists on appetite and weight in obese baboons. a–d, Daily IV infusion of 2 mg/kg of NT4 for 25 days into obese baboons (baseline body weight 20–30 kg, n = 3 per group) lead to a reversible increase in food intake (a) and body weight (b). Dual X-ray absorptiometry (DEXA) scan revealed an increase in total fat (c), with no change in lean body mass (d). e–f, IV dosing twice a week with 2 mg/kg NT4 over a period of 6 weeks lead to weight gain (f) with no significant change in food intake (e) in obese baboons (n = 3 per group). g, A schematic model depicting the dual, anorexic and orexigenic axes of the TrkB signaling system.

To determine the generality and effectiveness of peripheral TrkB agonists as orexigenic signal in primates, adult obese female baboons were injected IV with 2 mg/kg of NT4 daily for 25 days. Despite being obese at baseline, all NT4-treated animals increased their daily food intake by 2 to 3-fold (Fig. 4a) and consumed the entire daily food allowance of 35 biscuits up to 18 days. When the food allowance was raised to 45 biscuits per day (day 19 through day 25), two of the animals still consumed the entire allowance (Supplemental Fig. S7a). The cumulative food intake during the period of NT4 treatment was increased by 2.5-fold (Supplemental Fig. S7b). The hyperphagic behavior subsided within 7 days after the last dose of NT4 (Fig. 4a). Daily infusion of NT4 also led to a 16% gain in body weight in these obese baboons, which took more than 30 days to revert to baseline level following treatment withdrawal (Fig. 4b). Consistent with the weight gain, these NT4 treated baboons exhibited significantly higher fasting serum leptin levels (9433±841 pg/mL, n = 3) than those in the control group (2707±1603 pg/mL, n = 3) after 3 weeks of treatment (P<0.05, Student's t-test). No significant change in the fasting serum levels of amylin, ghrelin, insulin, pancreatic peptide, PYY, IL-6 or TNF-α were observed (data not shown).

Dual X-ray absorptiometry (DEXA) scan revealed a profound effect of NT4 treatment on body composition (Fig. 4c and 4d). NT4 treated baboons had gained 1.65±0.31 kg (mean±SEM) while vehicle treated baboons lost 1.36±0.47 kg of total fat mass (Fig. 4c, P = 0.01, two-tailed Student's t test with Welch correction). There was no significant difference in lean body mass (Fig. 4d, P = 0.10) or in the bone mineral density (data not shown) between the groups. Therefore, peripheral activation of TrkB results in a potent orexigenic effect, leading to increase in body weight and fat mass even in nonhuman primates with pre-existing obesity.

### Pro-Obesity Effects of Peripheral NT-4 Injections Is Not Due to Receptor Desensitization

It was formally possible that chronic exposure to potent TrkB agonists could have led to TrkB receptor downregulation and hence a loss-of-function phenotype. We therefore examined the effect of intermittent, twice a week IV dosing with 2 mg/kg NT4 administration in the obese baboons over a period of 6 weeks. The treated animals gradually gained up to 7–8%, while the vehicle group lost 3–4% of body weight on average, resulting in a significant difference between these groups (Fig. 4f). No significant change in food intake, however, accompanied the weight gain (Fig. 4e).

The similar pro-obesity effect following either daily or twice a week dosing of NT4 (Figs. 4a–f), which in primates has an half-life of less then 30 minutes (data not shown), is incompatible with the hypothesis of TrkB receptor down regulation. Likewise, no consistent changes in TrkB receptor levels in the baboon peripheral white blood cells, that could have indicated receptor down regulation, were detected following 24 daily IV treatments with 2mg/kg NT4 (Supplemental Fig. S7c). Finally, consistent with these observations in primates, there is no evidence of TrkB tolerance, desensitization or resistance in any mouse models with any dosages of TrkB agonists we have tested (Fig. 1). These findings indicate that the pro-obesity effects of peripherally applied NT4 are not mediated by receptor down-regulation and that peripheral NT4 administration may directly reduce metabolic rate independent of its effect on food intake in the nonhuman primates.

## Discussion

The findings described here suggest the existence in the primates of a novel, peripherally accessible, orexigenic and pro-obesity TrkB axis that opposes the traditional, centrally located anorexigenic TrkB axis (Fig. 4g). Both arms of the system utilize the TrkB signaling pathway and the metabolic end result would be determined by the relative strength of differentially localized TrkB signals. To our knowledge, this represents the first example in primates of diametrically opposite metabolic and behavioral outcomes mediated by the same signaling pathway via spatial compartmentalization. The anatomical sites of action for the peripherally accessible, orexigenic TrkB signal are currently unknown, but may include the enteric nervous system, the pancreatic and gut neuro-endocrine system, the vagal nerve and/or the circumventricular organs.

The default state of the TrkB system in the whole body appears to be anorexigenic as indicated by the fact that rodents [2], [3], [5] or humans [6], [7] carrying a loss of function allele of BDNF or TrkB locus exhibited early onset obesity and hyperphagia. However, the anorexigenic TrkB stimulus, which is mediated by BDNF in the VMH, is under dietary control and was shown to be suppressed following fasting in mice [3]. Therefore, under a given physiological state, either the central anorexigenic or the peripheral orexigenic TrkB system could dominate the metabolic outcome dependent on the relative levels of receptor activation (Fig. 4g). The peripherally accessible, orexigenic TrkB axis is either absent in rodents or had been quantitatively masked by the more dominant, central anorexigenic TrkB axis, which in small animals may be more accessible to peripherally applied TrkB receptor agonists [13].

Interestingly, adult anorexia nervosa patients reportedly display lower than normal serum levels of BDNF while obese adults present higher than normal serum BDNF levels [14], [15], [16], [17]. Until now these findings had been interpreted to be a failed compensatory response of the central anorexigenic TrkB system analogous to that observed with the leptin system [18], [19]. Our data now raise the distinct possibility that eating disorders such as anorexia nervosa may be caused in part by the “sub-normal” levels of circulating BDNF and the resultant failure to activate the peripherally accessible orexigenic TrkB axis. Conversely, obesity may be due in part to the “supra-normal” levels of peripheral BDNF which over-activate the orexigenic TrkB signaling. Significant amounts of BDNF are present in the platelets [20] and the pituitary gland [21], providing potential sources of TrkB agonists for peripheral regulation of the orexigenic TrkB axis. If true, selective activators of the peripherally accessible orexigenic TrkB site would represent novel therapeutic agents for anorexia and cachexia while selective inhibitors of this system may find utility in the treatment for hyperphagia and obesity.

## Methods

Additional details of materials and methods used are provided in the Supplemental Methods S1.

### Proteins

Recombinant human BDNF protein was purchased from Peprotech (Rocky Hill, New Jersey). Recombinant human NT4 protein was purified and refolded from an *E. coli* culture engineered to over-express NT4, using standard procedures (see Supplemental Methods S1). TrkB agonist antibodies were generated using the entire extracellular domain of human TrkB protein as the immunogen in Balb/c mice to generate hybridomas (see Supplemental Methods S1).

### Animal studies

All animal experiments were conducted according to the protocols approved by the IACUC of the respective institutions (see Supplemental Methods S1). Details of various routes of compound administration, measurement of food consumption and body weight were described in Supplemental Methods S1. Through out this paper, “Day 1” of a given study denotes the day when the first dose of a therapeutic agent was given.

### Statistics

All data and graphs were expressed in mean±SEM. Statistical analyses were performed by using PRISM (GraphPad Software Inc., San Diego, CA). Unless otherwise specified, all animal time course studies were analyzed by two-way ANOVA followed by Bonferroni post tests if and only if the overall P value is less than 0.05. The symbol * denotes P<0.05, ** P<0.01 and *** P<0.001 for pairwise comparisons by Student's t-test, by Dunnett's post test (one-way ANOVA) or by Bonferroni post tests (two-way ANOVA).

## Supporting Information

Supplementary Figure S1 (0.15 MB DOC)

Supplementary Figure S2 (0.14 MB DOC)

Supplementary Figure S3 (0.11 MB DOC)

Supplementary Figure S4 (0.15 MB DOC)

Supplementary Figure S5 (0.16 MB DOC)

Supplementary Figure S6 (0.15 MB DOC)

Supplementary Figure S7 (0.20 MB DOC)

Supplementary Table S1 (0.05 MB DOC)

Supplemental Methods S1 (0.06 MB DOC)
