# Supplementary material for: Appetite Enhancement and Weight Gain by Peripheral Administration of TrkB Agonists in Non-Human Primates
Source: PLoS One. 2008 Apr 2;3(4):e1900. doi: 10.1371/journal.pone.0001900 (PMC2270901; doi:10.1371/journal.pone.0001900)
Supplement: Supplementary Figure S1 — (0.15 MB DOC) [file pone.0001900.s001.doc]

**Figure S1.**  Potential ligand receptor interactions were characterized by BIAcore. The global kinetic parameters of each interaction are rank listed in Supplementary Table 1. Most interactions were characterized by fast, diffusion-limited on rates, which were beyond the resolution of BIAcore (k_on_~1e7 1/Ms). Off rates were more variable: for example, interactions of mature NGF with TrkA had very slow off rates, (T_1/2_>1hour), whereas the NT3/TrkA interaction decayed within seconds (T_1/2_=9sec). The TrkB mAb-Fab/TrkB interaction had a biphasic off rate, so only the initial phase of decay was fit to the model. Receptors were intentionally coated at low levels on the chip in order to space them far enough apart such that dimeric ligands could not bridge *in cis* between adjacent receptor molecules. By promoting conditions where ligands were forced to bind via only one of their two available binding sites, we minimized avidity effects in these measurements.

Lin et al. Supplemental Figure S1

**NGF**

**NT4**

**NT3**

**BDNF**

**TrkB**

**mAb**

**Fab**

Time (s)

Response (RU)

***TrkA***

***TrkB***

***TrkC***

***p75***

*No binding*

*No binding*

*No binding*

*No binding*

*No binding*

*No binding*

*No binding*

*No binding*

**TrkBmAb/rHu-TrkA TrkBmAb/rHu-TrkB TrkBmAb/rHu-TrkC TrkBmAb/rHu-P75**
