# Supplementary material for: Appetite Enhancement and Weight Gain by Peripheral Administration of TrkB Agonists in Non-Human Primates
Source: PLoS One. 2008 Apr 2;3(4):e1900. doi: 10.1371/journal.pone.0001900 (PMC2270901; doi:10.1371/journal.pone.0001900)
Supplement: Supplementary Figure S2 — (0.14 MB DOC) [file pone.0001900.s002.doc]

**Figure S2.**  The TrkB agonist antibody was chosen from a panel of antibodies that selectively bind TrkB.

**a.** Four TrkB-specific mouse monoclonal antibodies (38B8, 37D12, 36D1 and 23B8) were tested on E15 mouse nodose neuronal culture to screen for TrkB agonist activity. The nodose neuron cultures were prepared as previously described (Davies et al., 1993) and the number of surviving neurons were counted after 48 hours *in vitro* culture in the presence of varying concentrations of each TrkB antibody. While all four antibodies exhibited some bioactivity in supporting nodose neuronal survival, 38B8 is by far the most potent one.

**b.** A single bolus injection of TrkB antibody 38B8 (5mg/kg, IP, N=8), but not 37D12 (30mg/kg, IP, N=8) significantly reduced body weight than the control IgG (5mg/kg, IP, N=9) in adult female C57BL/6 mice. A higher dose of 37D12 was used in this study because this antibody has a lower intrinsic potency than 38B8 in the nodose neuronal assay. Arrow indicates the time of the bolus injection.

**c.** A single bolus injection of TrkB antibody 38B8 (5mg/kg, IP, N=8), but not 23B8 (30mg/kg, IP, N=8) significantly reduced body weight than the Vehicle (IP, N=9) in adult female C57BL/6 mice. A higher dose of 23B8 was adopted here also because this antibody has a lower intrinsic potency than 38B8 in the nodose neuronal assay. Arrow indicates the time of the bolus injection.

Lin et al. Supplemental Figure S2

a

b

c
