# Supplementary material for: Appetite Enhancement and Weight Gain by Peripheral Administration of TrkB Agonists in Non-Human Primates
Source: PLoS One. 2008 Apr 2;3(4):e1900. doi: 10.1371/journal.pone.0001900 (PMC2270901; doi:10.1371/journal.pone.0001900)
Supplement: Supplementary Figure S3 — (0.11 MB DOC) [file pone.0001900.s003.doc]

**Figure S3.** Subcutaneous (SC) administration of NT4 leads to gain in appetite, fat deposits and weight in lean cynomolgus monkeys. **a-c**, Daily treatment of non-obese, adult females, Cynomolgus (body weight 3-5 kg) with 2 mg/kg/day NT4 SC (n=3 per group) for 21 days led to a significant increase in daily food intake **(a)**, cumulative food intake **(b)** and body weight **(c).**

Lin et al. Supplemental Figure S3

**a**

**b**

**c**
