# Supplementary material for: Appetite Enhancement and Weight Gain by Peripheral Administration of TrkB Agonists in Non-Human Primates
Source: PLoS One. 2008 Apr 2;3(4):e1900. doi: 10.1371/journal.pone.0001900 (PMC2270901; doi:10.1371/journal.pone.0001900)
Supplement: Supplementary Figure S4 — (0.15 MB DOC) [file pone.0001900.s004.doc]

**Figures S4.** Blood electrolytes, biochemistry and complete blood cell counts were not significantly altered by daily IV administration of 2 mg/kg NT4 in lean, adult female cynomolgus monkeys over 30 days (n=3 per group). This study was the same one described in Figs. 3a-d.

Lin et al. Supplemental Figure S4

**a e**

**b f**

**c g**

**d** **h**
