# Supplementary material for: Appetite Enhancement and Weight Gain by Peripheral Administration of TrkB Agonists in Non-Human Primates
Source: PLoS One. 2008 Apr 2;3(4):e1900. doi: 10.1371/journal.pone.0001900 (PMC2270901; doi:10.1371/journal.pone.0001900)
Supplement: Supplementary Figure S7 — (0.20 MB DOC) [file pone.0001900.s007.doc]

**Figure S7.** Daily IV infused of 2 mg/kg of NT4 for 25 days into obese baboons (body weight 20-30 kg, n=3 per group) lead to increase in daily food intake **(a)** and cumulative food intake **(b)**. Each individual animal was color coded by their treatment group (Vehicle group in green shades, NT4 group in red shades) and by Animal ID# in both **a** and **c**. **c**. The peripheral white blood cells in buffy coat were collected and prepared 24 hours after the final IV dose. The total cell lysates were probed with either polyclonal TrkB antibody (upper panel) or a house keeping protein GAPDH antibody (lower panel). No significant change was evident in the total TrkB protein level between NT4 and Vehicle treated samples.

Lin et al. Supplemental Figure S7

**a**

**b c**

**FOOD INTAKE**


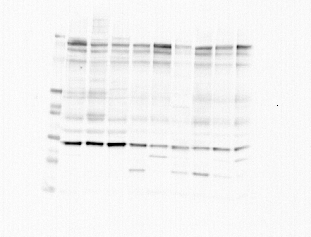


**NT4**

**Vehicle Vehicle**

**NT4**

TrkB

**GAPDH**


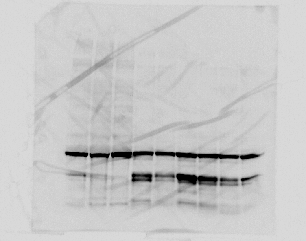


Treatment Animal ID

1x3873 1x4246 6311

7862
