# Supplementary material for: Appetite Enhancement and Weight Gain by Peripheral Administration of TrkB Agonists in Non-Human Primates
Source: PLoS One. 2008 Apr 2;3(4):e1900. doi: 10.1371/journal.pone.0001900 (PMC2270901; doi:10.1371/journal.pone.0001900)
Supplement: Supplementary Table S1 — (0.05 MB DOC) [file pone.0001900.s008.doc]

**Supplementary Table 1**: The binding specificity and affinity between the Trk receptors and ligands as determined by global kinetic analysis on BIAcore. The raw data were shown in Supplemental Fig S1. The interactions are ranked from high to low affinity (by K_D_). “Mean Average ± Standard Deviation” are given for those interactions that were analyzed in two independent experiments. TrkBmAb denotes the Fab fragement of the TrkB specific agonist antibody. The Fab fragment of TrkB mAb was used here for kinetic study to reduce the avidity effect due to the bivalency of the full antibody. “*” denotes that the fastest on-rate measurable by the Biacore 3000 platform is in the order of 1e7 1/Ms, which approaches the diffusion limit. We used this as a cut-off value for very fast onrates. “NB” denotes that “No Binding” was detected for certain ligand/receptor pairs.

| **Ligand/Receptor** | **k_on_ (1/Ms)** | **k_off_ (1/s)** | **K_D_ (nM)** |
| --- | --- | --- | --- |
| **NGF/TrkA** | (1.0 ± 0.5)e^7^* | (1.88 ± 0.02)e^-4^ | 0.018 ± 0.009 |
| **BDNF/TrkB** | 3.82e7* | 1.3e-3 | 0.034 |
| **NT4/TrkB** | 1e7* | (2.0 ± 0.2)e^-3^ | 0.20 ± 0.02 |
| **NT3/TrkC** | 1e7* | 0.0151 | 0.9 |
| **NGF/p75** | (2.8 ± 0.3)e^7^ | (4.9± 0.5)e^-2^ | 1.7 ± 0.2 |
| **NT3/p75** | 1e7* | 0.164 | 1.7 |
| **BDNF/P75** | 8.38e6 | 0.0174 | 2.1 |
| **NT3/TrkB** | 1e7* | (4.4 ± 0.8)e^-2^ | 4.4 ± 0.8 |
| **NT4/TrkA** | (2.1 ± 0.6)e^6^ | (4 ± 2)e^-2^ | 17 ± 11 |
| **NT3/TrkA** | (4.4 ± 1.0)e^6^ | (8 ± 2)e^-2^ | 18 ± 6 |
| **TrkBmAb /TrkB** | 2.03e5 | 7.91e-3 | 39 |
| **NT4/p75** | 3.45e6 | 0.305 | 88 |
| **NGF/TrkB** | - | - | NB |
| **NGF/TrkC** | - | - | NB |
| **BDNF/TrkA** | - | - | NB |
| **BDNF/TrkC** | - | - | NB |
| **NT4/TrkC** | - | - | NB |
| **TrkBmAb /TrkA** | - | - | NB |
| **TrkBmAb /TrkC** | - | - | NB |
| **TrkBmAb /p75** | - | - | NB |
