# Supplementary material for: Appetite Enhancement and Weight Gain by Peripheral Administration of TrkB Agonists in Non-Human Primates
Source: PLoS One. 2008 Apr 2;3(4):e1900. doi: 10.1371/journal.pone.0001900 (PMC2270901; doi:10.1371/journal.pone.0001900)
Supplement: Supplemental Methods S1 — (0.06 MB DOC) [file pone.0001900.s009.doc]

**Supplemental Methods**

**Recombinant Proteins.** Recombinant human BDNF was purchased from PeproTech Inc. (www.peprotech.com, catalog # 450-02). Recombinant human NT-4 protein was purified from an *E. coli* culture engineered to over-express NT-4, using a modification of published procedures (De Young, 1999; Zheng, 1995). Briefly, *E coli* cell paste was suspended at a concentration of 100 mg/mL in Tris/EDTA buffer (20 mM Tris, 5 mM EDTA, pH8.0) and disrupted by homogenization (3 passes at 660 bar). NT-4 in insoluble inclusion bodies was then harvested by centrifugation. This insoluble fraction was then solubilized in 6M urea, 25 mM DTT. Polyethyleneimine was added to 0.2% and the sample was clarified by centrifugation. This clarified supernatant was applied to a DEAE-Sepharose FF column (GE Healthcare, Piscataway, NJ), which had been equilibrated with 20 mM Tris, 6 M Urea and 10 mM DTT, pH 8.0. The pH of the non-bound flow-through fraction was adjusted to 5.0 with glacial acetic acid and this fraction was applied to an SP-Sepharose FF column (GE Healthcare, Piscataway, NJ) which had been equilibrated with 20 mM NaAcetate, 6 M Urea, pH 5.0. NT-4 was eluted from this column by washing the column in 20 mM NaAcetate, 6 M Urea, 0.5 M NaCl, pH 5.0. The NT-4 in the eluate was greater than 95% pure. The protein concentration of the eluate was adjusted to 10 mg/mL and the buffer exchanged to 0.2 M Tris, 4M GuHCl, 5 mM DTT, pH 8.3 by ultrafiltration /diafiltration on cellulose acetate membranes (Millipore). The NT-4 was then refolded by first adding oxidized glutathione to 20 mM then adding 19 volumes of 100 mM Tris, 20 mM Glycine, 1M GuHCl, 15% PEG 300, pH 8.3, and then adding cysteine to 3 mM. This solution was then saturated with nitrogen and was stirred at 4˚C for 16-18 hours. The progress of refolding was monitored by RP-HPLC. When refolding was determined to be complete, the protein was concentrated approximately 10-fold and then buffer exchanged to 10 mM Na Acetate pH 4.0. NT-4 was diluted to 0.5 mg/ml and 0.750 M NaCl was added. This solution was then applied to a Phenyl-650M HIC column (Tosoh, Tokyo, Japan). The resin was equilibrated with 10 mM Na Acetate pH 4.0, and 2.5 M NaCl. The protein was washed with 10 mM Na Acetate pH 4.0, and 0.5 M NaCl. NT-4 was eluted with 10 mM Na Acetate pH 4.0. Protein was concentrated to approximately 3 mg/mL and tested for purity, stability, and endotoxin levels. LAL assay (Charles River-Endosafe-PTS, Wilmington, MA) resulted in <3.5 EU/mg after HIC purification.

**TrkB Agonist Antibodies.** Monoclonal, TrkB-specific agonist antibodies were generated by immunizing Balb/C mice with the entire extracelluar domain of recombinant human trkB protein following the published RIMMS, i.e. Rapid IMmunization at Multiple Sites, protocol (Kilpatrick et al, 1997). Briefly, 10 ug of the entire extracellular domain of human TrkB, mixed with alum and Ribi adjuvant, was used to immunize each Balb/c mice subcutaneously at six different sites including the footpads and the base of the tail on Day 1. Another 8 ug of the antigen was injected intravenously on Day 2, followed by subcutaneous injections of additional 8 ug of the antigen at multiple sites on Day 4, 7 and 9. Splenocytes were collected on Day 14 and fused with the myeloma cell line P3x63Ag8.653 (ATCC, Manassas, VA) using polyenthylene glycol. Hybridomas were screened for trkB binding by ELISA and then for trkB agonist activity by cell based tyrosine kinase phosphorylation (Sadick, 1997) as well as by a neuronal survival assay using the mouse embryonic nodose ganglia (Davies, 1993). The ascites obtained from a single Balb/C mouse was purified batchwise with protein A resin. Approximately 1 ml of resin equilibrated with PBS was incubated with ascites fluid overnight at 4^o^C with gentle agitation. The resin was spun down and washed twice with PBS. Antibody was eluted from the resin using 5 volumes of 50 mM sodium citrate-phosphate buffer pH 3. Eluates were immediately neutralized with 1M hepes buffer pH 7, dialyzed into PBS, then concentrated and sterile filtered.

A panel monoclonal antibodies were identified that bind TrkB, but not other neurotrophin receptors TrkA, TrkC or p75 (for example, see Supplemental Table 1 and Supplemental Figure S1). Eventually four such TrkB-specific monoclonal antibodies were found to be capable of both increasing TrkB receptor phosphorylation in a CHO cell line stably expressing human TrkB (Tsao et al. Supplemental Info) and supporting the survival of embryonic day 15 mouse nodose ganglion neurons over 48 hours in vitro (Supplemental Figure 2a). We found that antibody 38B8 is a much more potent TrkB agonist than others (36D1, 23B8 or 37D12) as shown by their respective ability to support embryonic nodose neuron survival (Supplemental Figure S2a). Next we generated large quantities for three of these antibodies (38B8, 37D12 and 23B8) to test their systemic effect in the context of whole animals. These antibodies were administered systemically to the lean, adult female C57BL/6 mice to determine their effects on body weight. A single bolus injection of antibody 38B8 at 5mg/kg (N=8) significantly reduced body weight by 10% over 7 days (Supplemental Figure S2b-c). By contrast, neither antibody 37D12 (30 mg/kg, N=8, Supplemental Fig S2b) nor 23B8 (30 mg/kg, N=8, Supplemental Fig S2c) affect the body weight relative to the control IgG (N=9, Supplemental Fig S2b) or Vehicle (N=9, Supplemental Fig S2c). Therefore, there appears to be a rough correlation between TrkB agonist potency defined neuronal survival assays and the *in vivo* activity on body weight. Since all of the TrkB and control antibodies were tested for Endotoxin levels as part of the quality control and assurance prior to in vivo studies, it is very unlikely that LPS/Endotoxin contamination in the antibody preparations could contribute to the observed metabolic effects. Because of the superior TrkB agonist potency of the antibody 38B8, we used this antibody in all the other studies described in this work. For clarity and ease of reading, we called 38B8 “the monoclonal TrkB agonist antibody” through-out the main text.

The TrkB agonist antibody (i.e. 38B8) was shown to block the interaction between TrkB and its natural ligands, BDNF and NT4 (data not shown), indicating that this TrkB agonist antibody occupies a binding site on TrkB molecule that at least partly overlaps with the native ligand docking site.

**Protein interaction.** Interaction analysis was performed at 25ºC using a BIAcore 3000 instrument equipped with a CM5 sensor chip (BIAcore AB, Uppsala, Sweden). HBS-EP running buffer (used for the immobilizations) was purchased from BIAcore, along with amine-coupling reagents (NHS, EDC, acetate pH4.5, and ethanolamine). Fc fused recombinant human receptors (TrkA, TrkB, TrkC, and p75) and native ligands (NGF, BDNF, NT4/5, and NT3) were either purchased from R&D systems, Peprotech or prepared in-house.

*Immobilization of receptors.* Receptors (3 per chip) were immobilized onto CM5 sensor chips at *low* levels (typically 500RU, or 4fmol/mm2), leaving one flow cell unmodified (per chip) to serve as a reference surface. A standard amine-coupling protocol was used in HBS-EP running buffer at 5uL/min and involved three steps. First, flow cells were activated with a 7-min injection of a freshly prepared mixture of 200mM EDC in 50mM NHS; this step converted the chip’s carboxylic acid groups to reactive esters. Next, receptors were diluted to ~10ug/mL in 10mM sodium acetate buffer at pH4.5 and coupled to the chip until the desired level had been reached. Finally, excess reactive esters were blocked with a 7-min injection of 1M sodium ethanolamine.HCl pH8.5.

*Analysis of ligands.* Ligands (NGF, BDNF, NT4/5, and NT3) were diluted serially in running buffer (10mM Hepes pH7.4, 150mM (NH4)_2_SO4, 1.5mM CaCl2, 1mM EGTA, and 0.005% v/v Tween-20) to concentrations spanning 0.08-250nM in 5fold increments. The Fab fragment of antibody TrkB agonist antibody (TrkB mAb) was diluted from 0.7-480nM in 3fold increments. Samples were injected for 30sec at 100uL/min allowing dissociation times of up to 10mins. After each binding cycle, receptor surfaces were regenerated with a mildly acidic cocktail (10mM glycine.HCl pH4.0, 500mM NaCl, and 20mM EDTA). Duplicate injections confirmed that the assay was reproducible. Kinetic rate constants (kon and koff) were determined by fitting the binding data globally to a mass transport model. Affinities were calculated from their ratio (KD=koff/kon).

***In vivo* rodent studies.** All experiments using mouse models were conducted according to the protocols approved by the Institutional Animal Care and Use Committee (IACUC) of Rinat Neuroscience. All animals were housed in a temperature and humidity controlled room with a 12 hour light/dark cycle, with ad libitum access to water and food unless otherwise specified for the pair-fed study. The high fat diet-induced obesity (DIO) mice were produced as follows: Male C57BL/6J mice (JAX West, West Sacramento, CA) were weaned at 4 weeks of age; immediately upon weaning they were put on 58% high fat diet (D12331i, Research Diets). The average body weight of these DIO mice at the onset of treatment as described in Figs 1a-b was 36.9±4.6g (mean±SD). The *db/db* mice (BKS.Cg-m+/+*Lepr^db^*/J strain) were obtained from Jackson Laboratory (Bar Harbor, ME). They were fed on the regular mouse diet (PicoLab Rodent Diet 20, containing 4.5-5.4% fat). Additional methodological details of rodent experiments were as previously described (Tsao et al., 2007).

***In vivo* non-human primate studies.** All non-human primate experiments were conducted according to the protocols approved by the Institutional Animal Care and Use Committee (IACUC) of the following institutions respectively: rhesus monkey studies at Northern Biomedical Research and at Alpha Genesis Inc. (AGI), cynomolgus monkey studies at AGI. and the baboon studies at Southwest Foundation of Biomedical Research.

*Intracerebroventricular delivery in rhesus monkeys*. Four male and four female rhesus monkeys were scanned by MRI to provide stereotaxic coordinates for the surgery and to screen for abnormalities. The animals were assigned to two study groups by body weight and sex. The monkeys were implanted in the left cerebral ventricle with a catheter terminating in a subcutaneous access port. After a surgical recovery period of at least 7 days the animals were dosed. All animals had physical (e.g. heart rate, body temperature, respiration, eyes, ears, skin and nails, oral cavity, abdominal palpation, lymph nodes, thoracic auscultation, gait, and disposition) and neurological examinations at least once pre-surgery, once post-surgery, and 4 hours post dose on dosing days. Test articles (NT4, BDNF, etc.) were diluted in PBS to the appropriate concentration and were injected in a bolus volume of 0.5 mL over a period of 2 minutes on the indicated dosing day. Forty monkey biscuits (PMI Certified Primate Diet #5048, Richmond, Indiana) were placed in each animal’s feeder each day. Daily food intake was measured by counting the number of monkey biscuits consumed the next morning.

In total, 8 animals successfully underwent surgery for MRI-guided ICV catheterization. After a period of acclimation, a series of dose escalation study was started for BDNF and NT4 in parallel. On Day 1, four animals (A-D, two males and two females) received a bolus of 30 ug BDNF and the other four animals (E-H, also two males and two females) received a bolus of 30 ug NT4 (right panel in Fig.2a). When their daily food intake levels returned to baseline levels, animals A-D received a bolus of 100 ug BDNF and animals E-H received a bolus of 100 ug NT4 on Day 8. Similarly animals A-D received a bolus of 300 ug BDNF and animals E-H received a bolus of 300 ug NT4 on Day 22. After several rounds of PBS flushing of the ICV catheter line over the next 4 weeks, four animals (A-D) completely returned to baseline level of food intake and were subjected to vehicle injection (left panel of Fig.2a).

*Cynomologus monkey studies.* The age range for the 24 female cynomolgus macaques (Macaca fasicularis) at the study start was 9-11 years (NT4 daily IV treatment study) and 2-4 years (NT4 daily SC treatment study and TrkB agonist antibody twice weekly IV study). Prior to being assigned to the study the animals were either 1) imported from China into the U.S., underwent CDC quarantine, and being housed at AGI for future sale or 2) they removed from the AGI breeding colonies. Throughout the study period the cynomolgus macaques were singly housed and maintained in accordance with the guidelines contained within the Guide for the Care and Use of Laboratory Animals (National Research Council, National Academic Press, Washington DC, 1996). Additionally the procedures for the use and care of the macaques were approved by the Alpha Genesis, Inc. Institutional Animal Care and Use Committee. Each animal was individually housed for the duration of the study. All animals were acclimated to the study room for at least 3 days prior to the study. Manual restraint and/ or a pole and collar system were used to restrain the animals for dosing and sample collection. The animals were acclimated to these restraints prior to the start of the study. The animals were fed a specific number of Teklad Nonhuman Primate Diet (8788) each morning and afternoon. Prior to feeding the uneaten biscuits from the previous feeding were counted and removed. The number of biscuits eaten by each animal was recorded twice daily. The animals were observed twice daily throughout the study for any clinical signs of illness and for evidence of a menstrual cycle. The animals were anesthetized with Ketamine HCl (5-10mg/kg) intramuscularly prior to body weight determination. Other procedures scheduled at the same time as a body weight measurement were done while the animal was sedated. Two dose routes (intravenous and subcutaneous) were utilized in these studies. The intravenous dosing was accomplished by clipping the hair over a peripheral vein (i.e. saphenous or cephalic), prepping the site aseptically, and placing an intravenous (Jelco style) catheter for extended infusions or a butterfly catheter for bolus infusions. The subcutaneous dosing was accomplished by dividing the area over the scapula and lower back into quadrants by clipping the hair in the shape of square in these areas. The area was the aseptically prepared. A needle and syringe of varying sizes were used to inject the test article subcutaneously. The site of the previous dose was inspected at the next dose time point to ensure that there was no residual swelling or irritation has occurred. The same dose site was not used in 2 sequential dosing time points. Whole blood samples were taken at multiple time points through out each study for pharmacokinetics, clinical chemistry, and complete blood counts. Whole blood samples were collected from the femoral veins in the inguinal region. The site was clipped of hair and swabbed with alcohol prior to whole blood collection with a syringe and needle or a vacutainer system. Whole blood samples collected for pharmacokinetics were placed in to SST vacutainer tubes. The samples were allowed to sit at room temperature for 30 minutes. The samples were then centrifuged in a refrigerated centrifuge at 2500rpms for 15 minutes; the serum was removed and placed into two approximately equal aliquots for clinical biochemistry analyses conducted at either Anilytics Inc. (Gaithersburg, MD) or Antech Diagnostics (Morrisville, NC). The serum was processed just as the pharmacokinetic samples were processed. The clinical chemistry analysis included: Glucose, Insulin, C-peptide, triglyceride, Free Fatty Acid, Total Cholesterol, LDL, HDL, Potassium, Sodium, Chloride, and C - reactive protein. The complete blood counts were conducted at either Anilytics Inc. (Gaithersburg, MD) or Alpha Genesis, Inc. (Yemassee, SC). Alpha Genesis, Inc. utilized the Drew Scientific Hemavet 950 to analyze the samples. The samples were collected into vacutainer containing EDTA as an anticoagulant and held at ambient temperature until analyzed.

*Obese baboon studies.* The obese baboons used are primarily a mixture of two subspecies, *Papio hamadryas anubis* and *Papio hamadryas cynocephalus* at Southwest Foundation of Biomedical Research, San Antonio, TX. Prior to the studies, animals were gang housed and fed *ad libitum* on a standard chow diet. Selected for the study were the spontaneously obese female baboons meeting the following criteria: (1) age between 12-20 years old, (2) past history of abnormal fasting glucose levels (> 126 mg/dl), (3) history of continuous weight gain in the past and (4) body weight greater than 20 kg at the study onset. The animals were assigned to the different treatment groups to ensure a balanced distribution of baseline body weight.

*IV delivery with a tethered catheter system in baboons.* A housing and tether system was designed to permit sampling of body fluids, chronic monitoring of physiologic parameters (e.g. blood pressure, heart rate), performance of species typical behavioral interactions (aggression, affiliation, reproduction, etc), physical exercise (work on a motorized treadmill), assessment of water and diet consumption, as well as feces and urine collection. The system provided primates with the opportunity to engage in species typical social behavior and thereby minimized conditions which have been identified as contributing to the development of abnormal behaviors associated with individual housing. The system consisted of two parts: (a) a specialized cage system for housing small social groups of primates and (b) a tether and indwelling catheter system. Each modular system permitted four adult baboons (Papio cynocephalus anubis) to be tethered and housed in a social group. Each cage was 2.44 x 2.44 x 1.22 m (L x W x H) and could be subdivided by means of woven wire wall partitions. The tether system consisted of a backpack, a cloth jacket, a stainless-steel flexible cable containing electrical cables and catheters, and a saline infusion pump mounted on top of the cage. The system provides laboratory primates with the ability to socially interact with other nonhuman primates (Cohelho and Carey 1990).

*Morphometry.* Body weight was measured to the nearest 0.1 kg, and height was measured to the nearest centimeter. The body mass index (BMI) was calculated as weight in kilograms divided by height in meters squared. The waist circumference was measured midway between the lowest rib and iliac crest. Body composition was assed using dual energy x-ray absorpteometry (DEXA) and bioimpedance, and all measurements were performed by an experienced technician.

*Metabolic and Biochemical variables.* Fasting glucose, insulin and C-peptide levels, HbA1c, total cholesterol, tryglycerides, HDL, LDL were determined in all animals. A liver function profile, including ALT, also was determined. All blood chemistries (glucose, lipids, liver panel) were determined using ACE reagents by ACE and the NexCT Clinical Chemistry Systems (Alfa Wassermann, Inc., West Caldwell, NJ). The liver function panel was obtained bichromatically at different wavelengths. Fasting glucose levels were measured by the glucose oxidase method. Free fatty acid concentrations were determined using the fluorometric method. Total cholesterol and triglyceride concentrations were assayed using enzymatic colorimetric assay. The HDL-cholesterol was measured in blood samples after precipitation of lipoproteins with dextran sulfate. The low-density lipoprotein cholesterol level was calculated from the levels of high-density lipoprotein cholesterol and total cholesterol levels.

*Western blots of baboon peripheral white blood cells*. Baboon peripheral white blood cells were collected as buffy coat preparations. Total proteins were extracted in 200μl of RIPA buffer at 4°C (Teknova, Inc., Hollister, CA), supplemented with Complete protease inhibitor cocktail tablets (Roche, Inc., Indianapolis, IN) and 2mM of the phosphatase inhibitor sodium orthovanadate (MP Biochemicals, LLC, Aurora, OH), by homogenization of WBC pellets in ice-cold glass Potter-Elvehjem tissue homgenizers (Bellco Glass, Inc., Vineland, NJ). After homogenization, lysates were centrifuged at 15,000 rpm for 30min at 4°C. Total protein concentrations of lysate supernatants were calculated using the Bicinchoninic Acid Protein Assay (BCA) from Pierce Biotechnology, Inc. (Rockford, IL). 30μg of total protein from each sample was loaded onto a NuPage 4-12% Bis-Tris acrylamide gel (Invitrogen, Inc., Carlsbad, CA) and transferred to PVDF membranes (Invitrogen, Inc., Carlsbad, CA) by Western blot. The blot was then probed with a trkB extracellular domain-specific antibody (anti-TrkB, Millipore, Inc., Billerica, MA), and detected using an HRP-conjugated anti-rabbit IgG, in combination with the SuperSignal West Femto detection kit (Pierce Bitotechnology, Inc., Rockford, IL). Another identical blot was probed with the rabbit polyclonal antibody Trk (C-14) sc-11 (Santa Cruz Biotechnology, Inc., Santa Cruz, CA) which yielded similar results for TrkB. As a control, 60μg of total protein from each sample was transferred to PVDF, and probed with an HRP-conjugated goat polyclonal IgG specific to GAPDH (V-18) (Santa Cruz Biotechnology, Inc., Santa Cruz, CA).

**REFERENCES**

Comuzzie AG, Cole SA, Martin L, Carey KD, Mahaney MC, Blangero J, VandeBerg JL. The baboon as a nonhuman primate model for the study of the genetics of obesity. Obes Res. 2003;11(1):75-80.

[Coelho AM Jr](http://www.ncbi.nlm.nih.gov/entrez/query.fcgi?db=pubmed&cmd=Search&itool=pubmed_Abstract&term="Coelho+AM+Jr"%5BAuthor%5D), [Carey KD](http://www.ncbi.nlm.nih.gov/entrez/query.fcgi?db=pubmed&cmd=Search&itool=pubmed_Abstract&term="Carey+KD"%5BAuthor%5D). A social tethering system for nonhuman primates used in laboratory research. [Lab Anim Sci.](javascript:AL_get(this, 'jour', 'Lab Anim Sci.');) 1990;40(4):388-94.

Davies AM, Lee KF, Jaenisch R. p75-deficient trigeminal sensory neurons have an altered response to NGF but not to other neurotrophins. Neuron. 1993 Oct;11(4):565-74

De Young LR, Schmelzer CH, Burton LE (1999) A common mechanism for recombinant human NGF, BDNF, NT-3, and murine NGF slow unfolding. Protein Sci 8:2513-2518.

[Kilpatrick KE](http://www.ncbi.nlm.nih.gov/sites/entrez?Db=pubmed&Cmd=Search&Term="Kilpatrick KE"%5BAuthor%5D&itool=EntrezSystem2.PEntrez.Pubmed.Pubmed_ResultsPanel.Pubmed_RVAbstractPlusDrugs1), [Wring SA](http://www.ncbi.nlm.nih.gov/sites/entrez?Db=pubmed&Cmd=Search&Term="Wring SA"%5BAuthor%5D&itool=EntrezSystem2.PEntrez.Pubmed.Pubmed_ResultsPanel.Pubmed_RVAbstractPlusDrugs1), [Walker DH](http://www.ncbi.nlm.nih.gov/sites/entrez?Db=pubmed&Cmd=Search&Term="Walker DH"%5BAuthor%5D&itool=EntrezSystem2.PEntrez.Pubmed.Pubmed_ResultsPanel.Pubmed_RVAbstractPlusDrugs1), [Macklin MD](http://www.ncbi.nlm.nih.gov/sites/entrez?Db=pubmed&Cmd=Search&Term="Macklin MD"%5BAuthor%5D&itool=EntrezSystem2.PEntrez.Pubmed.Pubmed_ResultsPanel.Pubmed_RVAbstractPlusDrugs1), [Payne JA](http://www.ncbi.nlm.nih.gov/sites/entrez?Db=pubmed&Cmd=Search&Term="Payne JA"%5BAuthor%5D&itool=EntrezSystem2.PEntrez.Pubmed.Pubmed_ResultsPanel.Pubmed_RVAbstractPlusDrugs1), [Su JL](http://www.ncbi.nlm.nih.gov/sites/entrez?Db=pubmed&Cmd=Search&Term="Su JL"%5BAuthor%5D&itool=EntrezSystem2.PEntrez.Pubmed.Pubmed_ResultsPanel.Pubmed_RVAbstractPlusDrugs1), [Champion BR](http://www.ncbi.nlm.nih.gov/sites/entrez?Db=pubmed&Cmd=Search&Term="Champion BR"%5BAuthor%5D&itool=EntrezSystem2.PEntrez.Pubmed.Pubmed_ResultsPanel.Pubmed_RVAbstractPlusDrugs1), [Caterson B](http://www.ncbi.nlm.nih.gov/sites/entrez?Db=pubmed&Cmd=Search&Term="Caterson B"%5BAuthor%5D&itool=EntrezSystem2.PEntrez.Pubmed.Pubmed_ResultsPanel.Pubmed_RVAbstractPlusDrugs1), [McIntyre GD](http://www.ncbi.nlm.nih.gov/sites/entrez?Db=pubmed&Cmd=Search&Term="McIntyre GD"%5BAuthor%5D&itool=EntrezSystem2.PEntrez.Pubmed.Pubmed_ResultsPanel.Pubmed_RVAbstractPlusDrugs1). (1997) Rapid development of affinity matured monoclonal antibodies using RIMMS. Hybridoma 16(4):381-9.

Sadick MD, Galloway A, Shelton D, Hale V, Weck S, Anicetti V, Wong WL (1997) Analysis of neurotrophin/receptor interactions with a gD-flag-modified quantitative kinase receptor activation (gD.KIRA) enzyme-linked immunosorbent assay. Exp Cell Res 234:354-361.

Tsao D, Thomsen H, Chou J, Stratton J, Hagen M, Loo C, Garcia C, Sloane DL, Rosenthal A and Lin JC. (2007) TrkB agonists ameliorate obesity and associated metabolic conditions in mice. *Endocrinology* [Epub ahead of print, 2007 Dec 6].

Zheng JL, Stewart RR, Gao WQ (1995) Neurotrophin-4/5, brain-derived neurotrophic factor, and neurotrophin-3 promote survival of cultured vestibular ganglion neurons and protect them against neurotoxicity of ototoxins. J Neurobiol 28:330-340.
